# Redox-based ion-gating reservoir consisting of (104) oriented LiCoO_2_ film, assisted by physical masking

**DOI:** 10.1038/s41598-023-48135-z

**Published:** 2023-11-29

**Authors:** Kaoru Shibata, Daiki Nishioka, Wataru Namiki, Takashi Tsuchiya, Tohru Higuchi, Kazuya Terabe

**Affiliations:** 1 Research Center for Materials Nanoarchitectonics (MANA), National Institute for Materials Science (NIMS), 1-1 Namiki, Tsukuba, Ibaraki 305-0044 Japan; 2 Department of Applied Physics, Faculty of Science, Tokyo University of Science, 6-3-1 Niijuku, Katsushika, Tokyo 125-8585 Japan

**Keywords:** Electronic devices, Information storage

## Abstract

Reservoir computing (RC) is a machine learning framework suitable for processing time series data, and is a computationally inexpensive and fast learning model. A physical reservoir is a hardware implementation of RC using a physical system, which is expected to become the social infrastructure of a data society that needs to process vast amounts of information. Ion-gating reservoirs (IGR) are compact and suitable for integration with various physical reservoirs, but the prediction accuracy and operating speed of redox-IGRs using WO_3_ as the channel are not sufficient due to irreversible Li^+^ trapping in the WO_3_ matrix during operation. Here, in order to enhance the computation performance of redox-IGRs, we developed a redox-based IGR using a (104) oriented LiCoO_2_ thin film with high electronic and ionic conductivity as a trap-free channel material. The subject IGR utilizes resistance change that is due to a redox reaction (LiCoO_2_ ⟺ Li_1−*x*_CoO_2_ + *x*Li^+^ + *x*e^−^) with the insertion and desertion of Li^+^. The prediction error in the subject IGR was reduced by 72% and the operation speed was increased by 4 times compared to the previously reported WO_3_, which changes are due to the nonlinear and reversible electrical response of LiCoO_2_ and the high dimensionality enhanced by a newly developed physical masking technique. This study has demonstrated the possibility of developing high-performance IGRs by utilizing materials with stronger nonlinearity and by increasing output dimensionality.

## Introduction

Reservoir computing (RC) is attracting attention as a fundamental technology for the future data society, which will require high-speed, high-performance information processing of big data^1^. RC is a computational framework suitable for machine learning of time-series data, and a high-speed learning model with low computational cost compared to deep learning because fewer parameters are updated during learning^2–7^. It uses a reservoir to convert time-series input into spatiotemporal patterns, and enables pattern analysis with simple learning algorithms. Physical reservoirs are reservoir implementations that use various physical systems. Various types of physical reservoirs have been reported, such as soft bodies, optical devices, spin torque oscillators, and memristors^4,8–27^. Nonlinearity, high dimensionality, and short-term memory are required for reservoirs to achieve high computational performance, which is the focus of the construction of physical reservoir systems^1–7^.

In addition to computation performance, size is important for the implementation of RC devices to integrated circuits. We have recently reported ion-gating reservoirs (IGR) that are particularly small, and thus suitable for integration^25,26^. IGR is a physical reservoir in the form of nanoionics-based transistors in which electrochemical phenomena are utilized to modulate channel resistance^28–36^, which function is useful to perform RC^25,26^. While there are two types of IGRs; redox-IGR and electric double layer (EDL)-IGR, the redox-IGR that uses Li_*x*_WO_3_ as a channel material showed lower computational performance and operating speed^26^. Recent investigations on the redox reaction of WO_3_ with Li^+^ have pointed out that, during cycled insertion and desertion of Li^+^ into WO_3_, some of the inserted Li^+^ is irreversibly trapped in the WO_3_ matrix^37–39^. This supports the contention that the relatively low performance of WO_3_ redox-IGR originates from irreversible Li^+^ trapping and that the computational performance is low due to loss of the echo state property, which is an important property of reservoirs^2^. This indicates that redox-IGR with alternative channel materials, that do not exhibit such irreversible Li^+^ trapping, can obtain higher computing performance^37–39^.

To enhance redox-IGR performance, we developed a redox-IGR using a (104) oriented LiCoO_2_ thin film, with high electronic and ionic conductivity, as a trap-free channel material. The subject LiCoO_2_ redox-IGR utilizes the resistance change that results from the redox reaction (LiCoO_2_ ⟺ Li_1−*x*_CoO_2_ + *x*Li^+^ + *x*e^−^) accompanying the insertion and desertion of Li^+^^40–42^. It was determined from the results of a nonlinear autoregressive moving average (NARMA) task, a typical benchmark to evaluate RC’s computational performance, that the normalized mean squared error (NMSE), which indicates the calculation error, was 0.054, which equates to 67% lower than that of WO_3_ redox-IGR (NMSE = 0.163), and that the operation speed was 4 times faster^26^. The significant improvement in computational performance was analyzed from the perspectives of the nonlinearity, high dimensionality, and short-term memory of the device. Short-term memory was measured by the device’s memory capacity (MC), which was found to be lower compared to the WO_3_ redox-IGR; the MC of the LiCoO_2_ redox-IGR = 2.36, while the MC of the WO_3_ redox-IGR = 3.57. Despite having a lower MC, the superior nonlinearity and high dimensionality of the LiCoO_2_ redox-IGR led to the improvement of its computational performance^26^. While physical reservoirs require nonlinearity, high dimensionality, and short-term memory, enhancement of nonlinearity and high dimensionality improves computing performance only in the case with sufficient short-term memory for a specific task. Gate voltage (*V*_G_) sweep measurements of the LiCoO_2_ redox-IGR showed a more nonlinear resistance change with respect to the *V*_G_, which was attributed to the nonlinear composition change of LiCoO_2_ resulting from the insertion and desertion of Li^+^^40–42^. The high dimensionality of the device was evaluated by measuring the correlation coefficients between the nodes used in the reservoir computation. In addition to the use of Li^+^ trap-free LiCoO_2_ as the channel material, a physical masking technique that was newly developed for the present study and which was implemented by drain voltage pulse trains, also significantly contributed to the enhance computation performance of the LiCoO_2_ redox-IGR. It was discovered that the physical masking can noticeably improve high dimensionality without modifying the device at all, which indicates the huge potential of the technique. Furthermore, it is possible that physical masking could be used for other physical reservoirs by employing other methods (light, magnetism, mechanical stimulation, etc.) as was done in the present study. This study demonstrates the possibility of developing high-performance RC devices by utilizing materials with stronger nonlinearity and by increasing output dimensionality.

## Results and discussion

### LiCoO_2_ redox-IGR device structure and electrical characteristics

**Figure 1:**
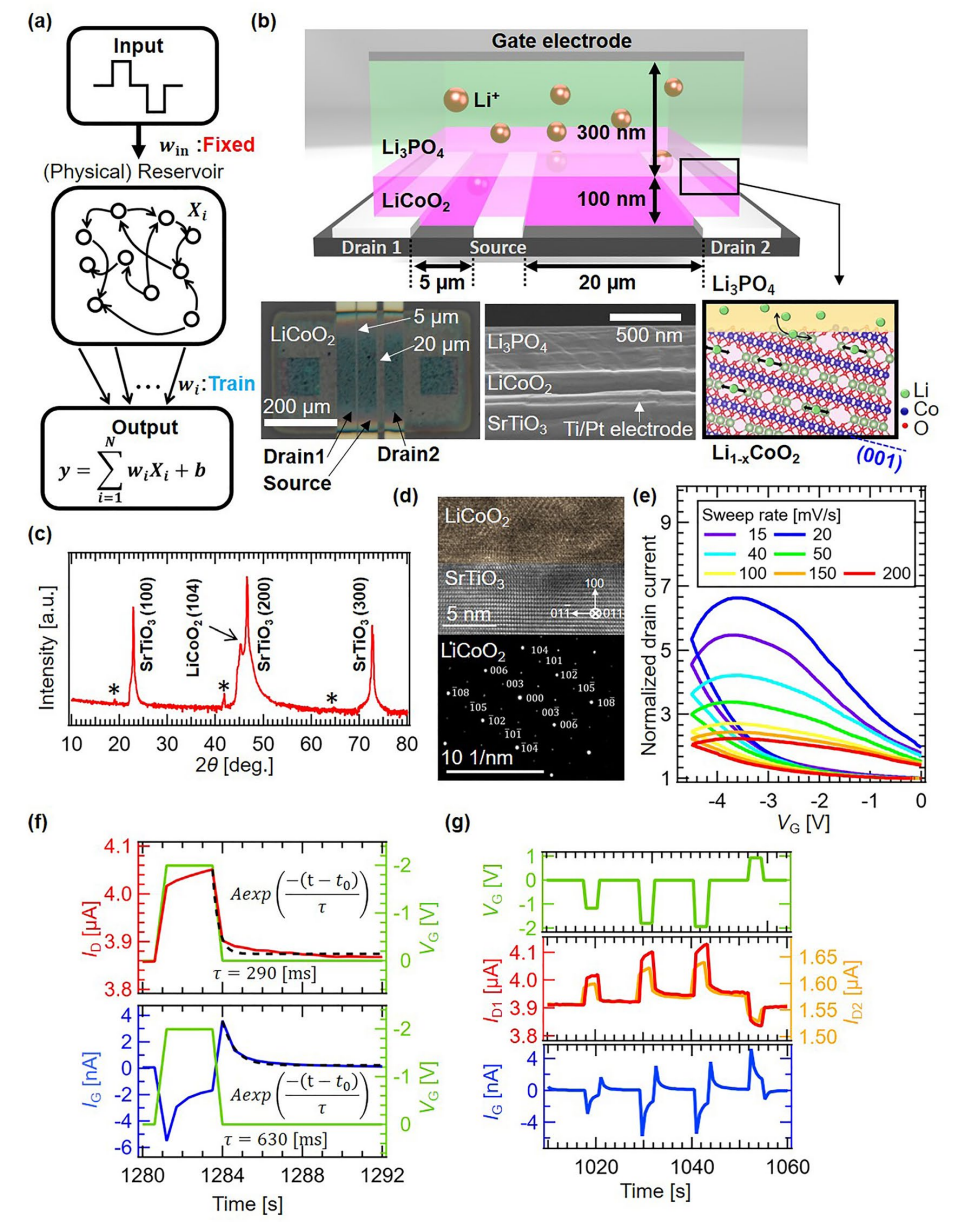
(**a**) General scheme of the subject reservoir computing system with (Physical) reservoir. *w*_in_ and *w*_i_ denote the input weight and read-out weight, respectively. (**b**) Schematic image of LiCoO_2_-based redox-ion-gating reservoir, cross-sectional SEM micrograph of a LiCoO_2_ redox-IGR, and insertion (desertion) of Li^+^ in (104) oriented LiCoO_2_. (**c**) XRD pattern of LiCoO_2_/SrTiO_3_. Stars mark the kβ diffraction peaks. (**d**) Cross-sectional TEM image and electron diffraction of LiCoO_2_/SrTiO_3_. (**e**) Normalized *I*_D_–*V*_G_ curve of the subject redox-IGR during *V*_G_ sweeping from 0 to − 4.5 V. (**f**) Changes in drain (Upper) and gate (Lower) currents when a single pulse of *V*_G_ is applied. (**g**) (Upper) *V*_G_ pulse stream input, (Middle) *I*_D1_ and *I*_D2_ response, and (Lower) *I*_G_ response during operation of the redox-ion-gating reservoir.

The general model of RC is shown in Fig. 1a. Time-series data are input to the reservoir to obtain the reservoir state vector^1^. The time series data input to the reservoir is transformed nonlinearly into a high-dimensional feature space as reservoir states *X*_*i*_ (*i* = 1, 2, …, *N*). In a full-simulation reservoir such as an echo state network, this nonlinear transformation is performed by a complex network with activation functions defined by sigmoid functions, etc., while in a physical reservoir, it is computed directly by the nonlinear dynamics inherent in the physical system^4,8–27^. To obtain the desired output, the readout weights *w*_*i*_ are trained by a simple algorithm, such as linear regression, and the reservoir output *y*(*k*) is obtained by a linear combination of the reservoir state *X*_*i*_(*k*) and the weights *w*_*i*_ as follows:

1 $$\begin{array}{c}y\left(k\right)=\sum_{i=1}^{N}{w}_{i}{X}_{i}\left(k\right)+b.\end{array}$$

Here, *N*, *k* and *b* are the reservoir size, discrete time step and bias, respectively.

A schematic of our LiCoO_2_ redox-IGR, implemented for physical reservoir computing, and cross-sectional scanning electron microscopy (SEM) micrograph of a LiCoO_2_ redox-IGR, are shown in Fig. 1b. Ti (5 nm)/Pt (35 nm) source and drain electrodes were deposited by electron beam evaporation on the surface of a SrTiO_3_ (100) substrate. LiCoO_2_ film (100 nm) was deposited by pulsed laser deposition (PLD) using an Nd:YAG laser operating at 266 nm wavelength, with an O_2_ gas fixed flow supplied at a rate of 5.4 sccm. The substrate temperature was kept at 600 °C during deposition. Li_3_PO_4_ (300 nm) and Si (20 nm) were deposited by the RF sputtering method at room temperature using Li_3_PO_4_ and Si targets, respectively, with a supply of pure Ar gas at a fixed flow rate of 10 sccm. Sputtering times were 180 min for Li_3_PO_4_ target and 8 min for Si target, respectively. And sputtering power and pressure were 50 W and 0.93 Pa for both Li_3_PO_4_ and Si targets. We chose the amorphous Li_3_PO_4_ because it owns compatibility with relatively high Li^+^ conductivity and stability. That is the reason why the amorphous Li_3_PO_4_ is widely used as a solid electrolyte for solid-state batteries^43,44^. Si was used for the gate electrode, since it is a promising material for the anodes of solid-state lithium batteries due to its high Li capacitance and low working electric potential^29,40^. A Pt current collector (50 nm) was deposited on the Si by electron beam evaporation. Two drain electrodes and a source electrode were fabricated so that the channel lengths were 5 and 20 µm, with a channel width of 500 µm as shown in lower left panel of Fig. 1b.

Figure 1c shows the XRD pattern for a LiCoO_2_ thin film on a SrTiO_3_ single crystal. The diffraction peak observed at 2$\theta$ of 45.24° is assigned to LiCoO_2_ (104)^45^ (JCPDS card no. 75-0532). Fabricated LiCoO_2_ thin film has no impurity phase, since there was no peak originating from other than (104). The lattice spacing calculated from the diffraction peak was 2.00 Å, which is in very good agreement with the bulk value of 2.00 Å^46^. Cross-sectional TEM observation of the LiCoO_2_/SrTiO_3_ interface shown in Fig. 1d was performed in order to obtain more detailed information on its crystallinity. Electron diffraction of the LiCoO_2_ shows that the LiCoO_2_ grew in 104 orientations, perpendicular to the substrate surface, which result is the same as for the XRD pattern. Furthermore, clear diffraction spots can be seen for directions parallel to the substrate surface, indicating that the LiCoO_2_ thin film has high crystallinity with aligned in-plane orientation.

By using a (104)-oriented Li^+^-hole mixed conduction LiCoO_2_ thin film as a channel, which exhibits high hole and ion conductivity^46^, we investigated the improvement of device operating speed and computational performance through the rapid and smooth insertion and desertion of Li^+^. By application of a *V*_G_, this IGR changes the channel resistance by inserting (deserting) Li^+^ into (from) the channel through the redox reaction shown below as Eq. (2):

2 $$\begin{array}{c}{\mathrm{LiCoO}}_{2}\Leftrightarrow {\mathrm{Li}}_{1-x}{\mathrm{CoO}}_{2}+x{\mathrm{Li}}^{+}+x{\mathrm{e}}^{-}.\end{array}$$

When a negative *V*_G_ is applied, Li^+^ are removed from the channel (i.e., *x* increases) and move through the solid electrolyte to the Si gate electrode shown in the lower panel of Fig. 1b. During the removal of Li^+^ from the channel, Co^3+^ is oxidized to Co^4+^ and electron holes are generated to maintain charge neutrality. This is accompanied by an increasing in the electrical conductivity of the Li_1−*x*_CoO_2_. This reaction in high quality LiCoO_2_ thin film is highly reversible^40–42,46^, so when a positive *V*_G_ is applied, the conductivity of the channel decreases as Li^+^ are inserted into the channel (i.e., *x* decreases). To measure the resistance modulation of the channel with respect to the *V*_G_, we measured the drain current (*I*_D_) during *V*_G_ sweeping.

The electrical characteristics of the fabricated device were analyzed at room temperature, in a vacuum chamber, using the source measurement unit (SMU) of a semiconductor parameter analyzer (4200A-SCS, Keithley). Materials in our devices were so stable that we could fabricate our devices in air, although we performed electrical measurements in vacuum for keeping our devices in the best condition. Such condition can be easily obtained by using encapsulation technology^47^, which has been well established for fabrication of integrated circuits (ICs) in commercial electronic devices. For example, transfer molding and compression molding using various types of resin (semiconductor encapsulant) are widely used to keep ICs in vacuum to protect them. Therefore, there is no severe limitation to practical applications. The normalized *I*_D_–*V*_G_ curve of the redox-IGR is shown in Fig. 1e. The *V*_G_ was swept from 0 to − 4.5 V and then back to 0 V at various sweep rates, ranging from 15 (slow) to 200 mV/s (fast). Nonlinearity in *I*_D_ change was confirmed from the *I*_D_ by the redox reactions shown in Eq. (2). These are associated with the insertion and desertion of Li^+^ that changes the channel resistance, and a clear hysteresis curve can be drawn at any sweep rate, which suggests that the device exhibits short-term memory, which in turn is a necessary function for reservoirs^1–7^. Generally, a dynamical system with a specific time constant shows a hysteresis when an external stimulation with a time constant, which is close to the one of the dynamical systems, is applied. In contrast, when the input is sufficiently faster than the time constant of the dynamical system, the response does not follow and does not show hysteresis. Also, when the input is sufficiently slow, the response corresponds to the steady state of the system and does not show hysteresis. Therefore, the extent of hysteretic behavior has a peak with respect to the speed of external stimulation. In the present case shown in Fig. 1e, the hysteresis of 20 mV/s is larger than the one of 15 mV/s because the sweep rate of 20 mV/s is closer to the peak discussed above.

Changes in drain and gate currents when a single pulse of gate voltage is applied are shown in the Fig. 1f. Each current relaxation was fitted with Eq. (3) as shown in black dotted lines.

3 $$\begin{array}{c}f\left(t\right)= {y}_{0}+A{\text{e}}{\text{x}}{\text{p}}\left(\frac{-\left(t-{t}_{0}\right)}{\tau }\right).\end{array}$$

Relaxation time $\tau$ was $290$ and $630\, \mathrm{ms}$, respectively. (The other fitting parameters and details of the fitting can be found in Supplementary Table S1). The drain and gate currents show a relaxation with respect to the pulse input, indicating that the device has short-term memory. Different drain and gate current relaxation times enhance the diversity of the nodes and improve computational performance. In particular, the gate current has a nonlinear and complex response to the *V*_G_ pulse, and the complex response is expected to lead to higher computational performance as in EDL-IGRs^25^. Our IGR utilized such nonlinear *I*_D_, *I*_G_ responses to Li^+^ insertion and desertion into LiCoO_2_ channel, driven by *V*_G_ input, as the nonlinear dynamics function that makes it possible for physical reservoirs to perform information processing.

The subject IGR is operated by a *V*_G_ pulse stream^25,26^. The time-course nonlinear response of *I*_D_ and the gate current (*I*_G_) outputs are shown in Fig. 1g. The upper panel of the figure shows the* V*_G_ pulse stream, which is the input from the IGR. The middle panel of the figure shows that different responses were obtained by using the two drain electrodes with different channel lengths. Upon application of the *V*_G_ pulse stream, the effective potential drops on the two channels are different due to different channel resistance, which is useful to enhance the diversity of the *I*_D_ response^25^. Since the *I*_G_ has a different shape and nonlinearity from *I*_D_, the *I*_G_ shown in the lower panel of the figure was also used in the reservoir calculation^26^. The behavior of the *I*_G_ is different from the *I*_D_, and the use of the *I*_G_ in the calculation is expected to enhance the high dimensionality of the reservoir and to significantly improve its computational performance.

Concerning the relatively slow operation speed, it can contribute to process a time-series data with slow change. In order to process time series data with a specific speed, response speed of the physical reservoir should cover the dynamics of the data. Therefore, in order to process a time-series data with slow change, the physical reservoir with slow response to input signal is needed. A typical example of that is the predication task of blood glucose level with slow change, which exhibits several ups and downs over several hours in a day^48^.

### Physical masking, for high dimensionality enhancement, implemented by drain voltage pulses

Masking is a pre-processing of input whereby the dimensionality of a physical reservoir system is effectively maximized^1,49^. In order to achieve high dimensionality, which is one of the characteristics required for reservoirs, the number of outputs (reservoir states) obtained from the reservoir for a given input must greatly exceed the input dimension. In system reservoirs, high dimensionality can be easily achieved by increasing the network size (number of nodes), but it is generally difficult to obtain a sufficient number of reservoir states physically in a physical reservoir due to limitations such as measurement probes^26^. Therefore, the virtual node method, which virtually considers the time evolution of the response to the input obtained from the physical system as spatially distinct nodes, is widely adopted in physical reservoirs. However, it is difficult to achieve sufficiently high dimensionality by simply using the transient responses of the physical system as different nodes in RC, because neighboring virtual nodes behave in a similar manner, and the effective number of nodes does not increase. Thus, by combining the input signal with a masked waveform containing fluctuations (masking), the virtual nodes behave differently from each other and high dimensionality can be improved. Masking is performed by introducing a (*N*_M_ × *Q*) mask matrix ***M*** for the *Q*-dimensional input signal ***u***(*k*), as follows:

4 $$\begin{array}{c}{\varvec{J}}\left(k\right)={\varvec{M}}\times {\varvec{u}}\left(k\right).\end{array}$$

**Figure 2:**
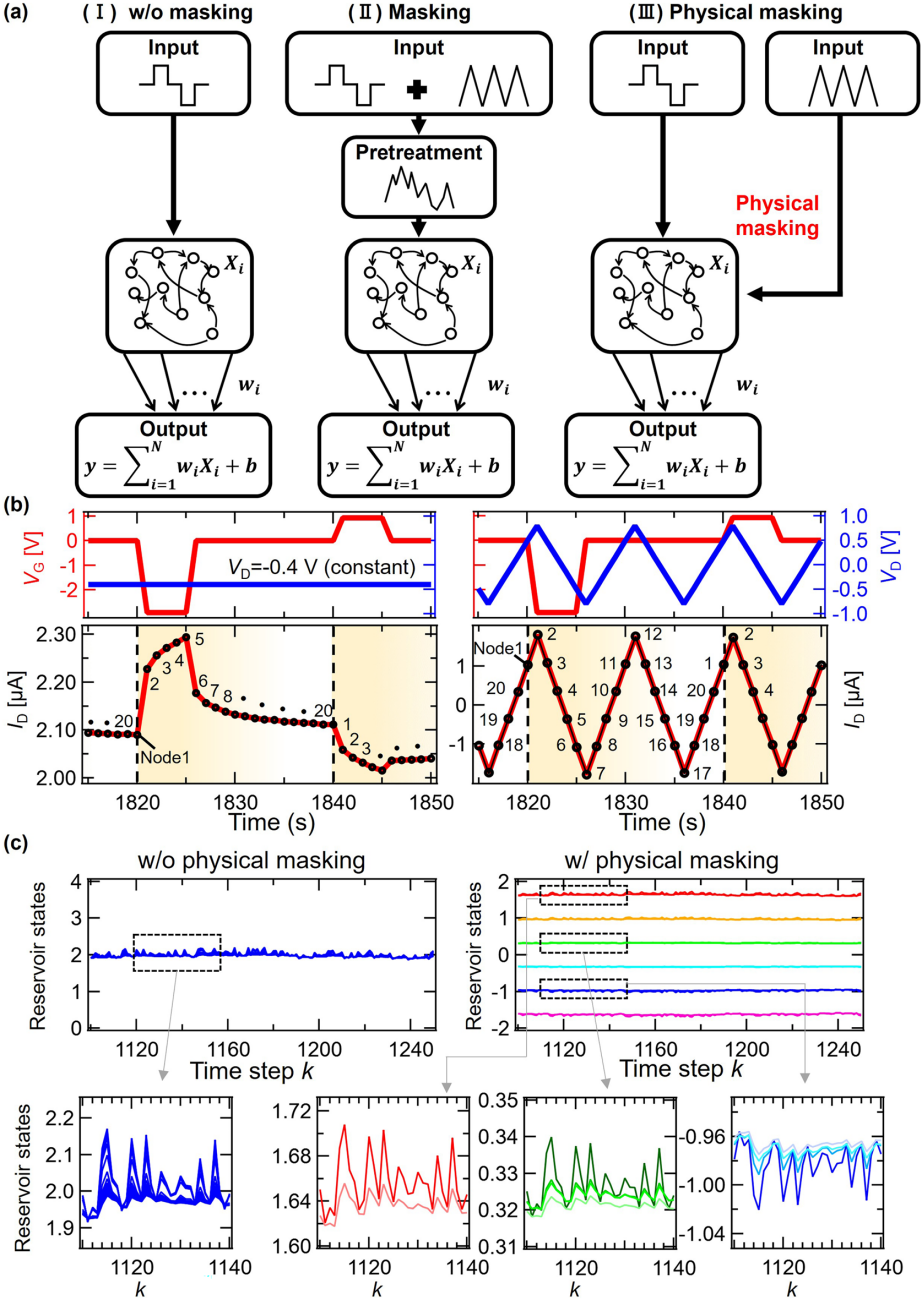
(**a**) (**I**) RC without masking, (**II**) RC with masking, and (**III**) RC with physical masking. **(b)** *V*_G_ and *V*_D_ input and *I*_D_ output w/o and w/ physical masking. **(c)** Reservoir state waveforms (*X*_1_,* X*_2_…, *X*_20_) w/o and w/ physical masking.

Here, *N*_M_ and ***J***(*k*) are the number of mask dimensions and the masked input signal, respectively; ***M*** is always fixed at times *k*, and a random number sequence or random bit sequence is used. For example, the masked input ***J***(*k*) is an *N*_M_-dimensional vector if *Q* = 1. When ***J***(*k*) is actually input to the physical system, these *N*_M_ elements are input to the physical system at a fixed time interval *θ*. Then, after completing input of the *N*_M_ masked input signals (i.e., after *θ* × *N*_M_ has elapsed in real time *t*), the next step of input ***J***(*k* + 1) is performed in the same manner. In this way, masked input is generated by synthesizing raw input with a specific mask waveform, including random ones, as a pre-processing of the input signal. By designing the mask matrix appropriately, and inputting ***J***(*k*) at time intervals that consider the time constant of the physical system (preferably shorter than the time constant of the physical system), interaction between neighboring virtual nodes is strengthened and the high dimensionality and nonlinearity are improved^21,49^. The left-hand and middle panels of Fig. 2a show RC without masking (left) and with masking (middle). When masking is used, time-multiplexing of the input is achieved and high dimensionality is enhanced. While masking generally leads to better computing performance, it creates an extra pre-processing calculation burden.

In the present study, a physical masking was developed and applied to the subject IGR to achieve high performance, as shown in the right-hand panel of Fig. 2a. By utilizing a structural feature of the IGR, multi-input terminals (i.e., gates and drains), a mask waveform can be directly input to the reservoir through the drain electrodes as drain voltage pulse trains, as shown in Fig. 2b. In this case, masking does not require pre-processing of the input and the masking is physically performed. Such is the physical masking we propose in this study. By utilizing a physical mask, the *I*_D_ response is changed from the one shown in the left-hand panel of Fig. 2b to the one shown in right-hand panel of Fig. 2b. Although the *I*_D_ response with physical mask seems to appear as a monotonous triangle wave, it does in fact generate reservoir states with excellent diversity. The reservoir states obtained from *I*_D_ responses (without and with physical masking) shown in Fig. 2b are compared to those in Fig. 2c. Without physical masking, reservoir states are concentrated in a narrow region from 1.92 to 2.17. Conversely, when physical masking is used, reservoir states are spread to six lines, each of which consists of diverse reservoir states. As indicated in the resulting clear differences in the versatility of reservoir states, physical masking significantly contributes to the enhancement of the computing performance of LiCoO_2_ redox-IGR, as well as to the inherent Li^+^ trap-free characteristic of LiCoO_2_, as shown in the following section.

### Solving a second-order nonlinear dynamic equation

**Figure 3:**
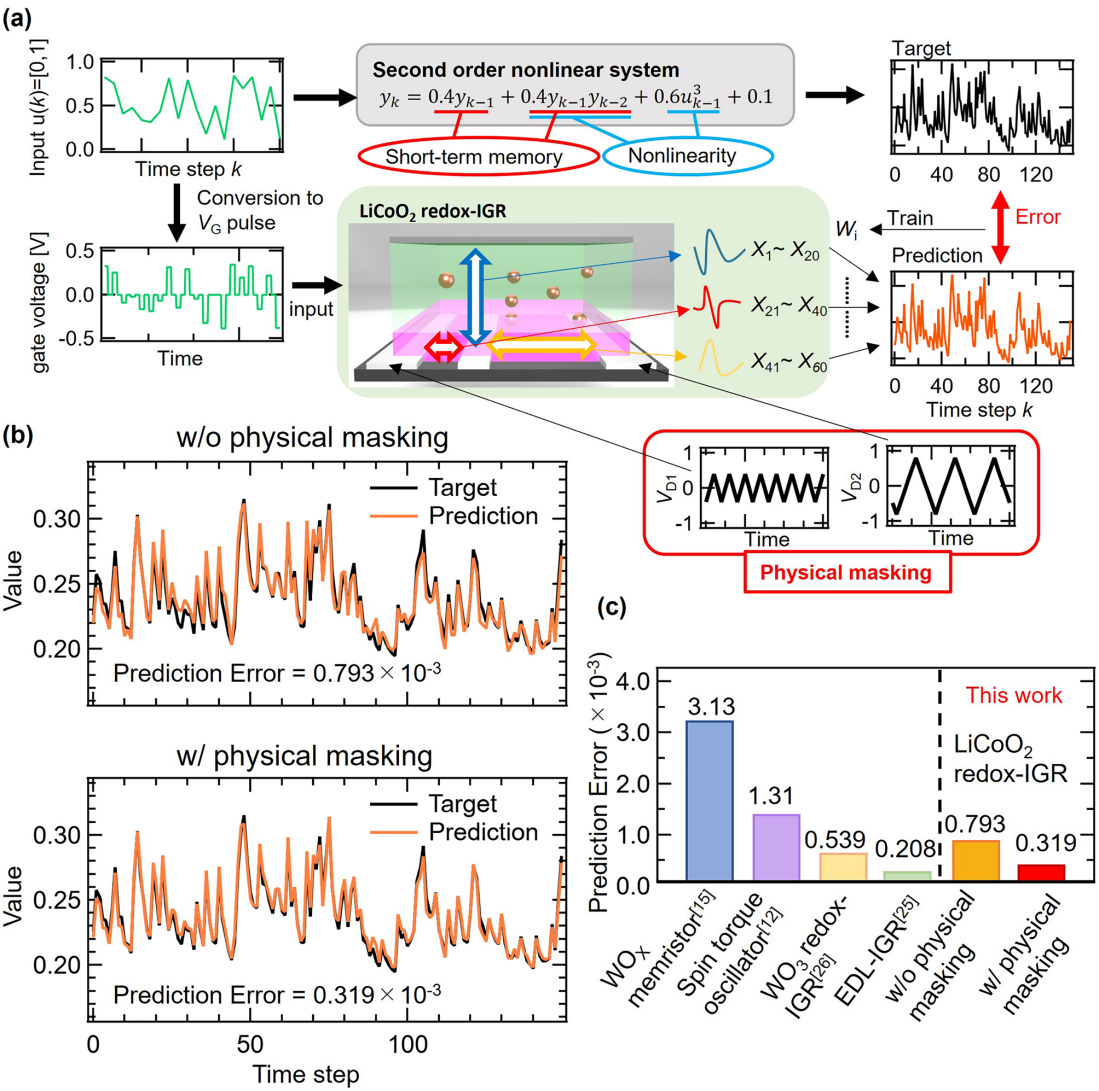
(**a**) Solving a second-order nonlinear dynamic equation task. (**b**) Target and prediction waveform of second-order nonlinear dynamics equation task w/o and w/ physical masking. (**c**) Performance comparison with other physical reservoirs.

In order to evaluate the effect of the subject physical masking on computational performance, we solved a second-order nonlinear dynamics equation task by a redox-IGR with *V*_D_ induced physical masking, as shown in Fig. 3a^12,15^. The target waveform *y*_t_(*k*) is generated by the second-order nonlinear dynamic equation shown in Eq. (5), which includes second-order nonlinearities and past data:

5 $$\begin{array}{c}{y}_{\mathrm{t}}\left(k\right)=0.4{y}_{\mathrm{t}}\left(k-1\right)+0.4{y}_{\mathrm{t}}\left(k-1\right){y}_{\mathrm{t}}\left(k-2\right)+0.6{u}^{3}\left(k\right)+0.1.\end{array}$$

Here, *u*(*k*) is a random input ranging from 0 to 0.5. The random input *u*(*k*) was converted to a voltage pulse streams with *V*_G_ of *V*_G_(*k*), a pulse period *T* of 10 s, duty rate *D* of 25%, and input to the gate of the IGR. *V*_G_(*k*) was linearly transformed from *u*(*k*) to a range of − 1 V to 2 V, as follows, with *V*_G_ = 0 V at the pulse interval as shown in the upper panel of Fig. 2b:

6 $$\begin{array}{c}{V}_{\mathrm{G}}\left(k\right)=2{V}_{\mathrm{ap}}u\left(k\right)+{V}_{\mathrm{offset}}.\end{array}$$

Here, *V*_ap_ (= 3 V) and *V*_offset_ (= − 1 V) are the amplitude and offset voltages, respectively. In addition to the drain currents obtained from two channels with different lengths, to achieve higher-dimensional reservoir states via the optimized physical structure, the gate currents with spikes were also used in the reservoir computing, as shown in the lower panel of Fig. 1f. This lead to enhanced high dimensionality due to the variety of reservoir states included^25,26^. These drain currents, corresponding to the reservoir state, were measured with the following two types of drain voltages: (1) *V*_D_ without physical masking: a constant *V*_D_ of 0.4 V and (2) *V*_D_ with physical masking: a stepped triangular wave *V*_D_ with a voltage range of − 0.4 V to 0.4 V, a period of *T*/4 for *V*_D1_ and − 0.8 V to 0.8 V, and a period of *T*/2 for *V*_D2_. The *I*_D_ response with and without physical masking is shown in the lower panel of Fig. 2b. While the *I*_D_ response exhibits a single relaxation-like behavior without physical masking, with physical masking, it appears as a stepped triangular wave similar to the applied *V*_D_ stream for physical masking. As will be clarified later, the output with such a triangular wave form includes sufficient variety. To further increase the higher dimensionality of the reservoir states, 20 current values per *V*_G_ pulse were obtained at virtual nodes, as shown in Fig. 2b. 20 reservoir states were obtained from each of the three current responses, so the reservoir size *N* of the redox-IGR was 60. The reservoir states ${X}_{i}\left(k\right)$ (i = 1,2,…,20) corresponding to *I*_D1_ are shown in Fig. 2c. When physical masking is not utilized (i.e., *I*_D_ responses were measured under constant *V*_D_), the behavior of these reservoir states is similar and low in diversity. On the other hand, when masking (i.e., *I*_D_ responses were measured with triangular wave *V*_D_ inputs) is used, the reservoir states are clearly diverse and achieve good high dimensionality. Such high-dimensionality will be discussed later.

The reservoir output is obtained by a linear combination of the readout weights ${w}_{i}$ which was trained by ridge regression^25^ and the reservoir state ${X}_{i}\left(k\right)$. The computational performance of the redox-IGR in this task was evaluated by ‘prediction error’, as follows:

7 $$\begin{array}{c}prediction\, error=\frac{\sum_{k=1}^{n}{\left[{y}_{t}\left(k\right)-y\left(k\right)\right]}^{2}}{\sum_{k=1}^{n}{\left[{y}_{t}\left(k\right)\right]}^{2}}.\end{array}$$

Here, *n* is the data length, *n* = 1100 for the training phase and *n* = 150 for the test phase. The initial 200 steps were excluded from the computation in order to wash out the initial state of the device. Figure 3b shows the predicted and target waveforms obtained without (upper panel) and with (lower panel) physical masks. The predicted waveform and the target waveform are in better agreement with each other with masking than without physical masking, and Eq. (5) is solved more correctly when physical masking is employed. Physical masking also reduced the training error by 65% (prediction error of 6.22 × 10^–4^ to 2.16 × 10^–4^) and the test error by 60% (prediction error of 7.93 × 10^–4^ to 3.19 × 10^–4^). This indicates that *V*_D_-induced physical masking is effective in improving the computational performance of redox-IGR. Also, to examine the effects of Physical masking, we performed additional experiments when two signal streams are applied to one gate electrode, and the same single stream is applied to the gate and drain electrodes. We solved a second-order nonlinear dynamics equation task. Applying two signal streams input to a single electrode is a general masking which is the pretreatment of input as shown in Fig. 2a(II) and the prediction error under the condition was $5.38\times {10}^{-4}$ which was better than the case without physical masking [prediction error: $7.93\times {10}^{-4}$ as shown in Fig. 3b], but worse than the case with physical masking [prediction error: $3.19\times {10}^{-4}$ as shown in Fig. 3b]. When the same inputs were applied to the gate and drain electrodes, the prediction error was $1.01\times {10}^{-3}$, which was worse than the case without physical masking. Details are explained in the Supplementary Information. According to the results, the physical masking shown as (III) in Fig. 3a was the best among them.

Figure 3c shows a comparison of performance with the physical reservoirs that have been reported to date^12,15,25,26^. Without physical masking, the computation performance was slightly lower than with WO_3_ redox-IGR, but with physical masking, the computation performance greatly exceeded WO_3_ redox-IGR, and the operating speed is also four times faster than that of WO_3_ redox-IGR^26^. By masking, the computational performance of the redox-IGR is further improved, making it comparable to that of EDL-IGR, which device exhibits high computational performance^25^. This result proves that masking overcomes the challenges of conventional redox-IGRs in terms of computational performance. The reasons for the significant improvement in computational performance compared with WO_3_ redox-IGR will be discussed in later sections.

### Solving a NARMA2 task

In addition to the second-order nonlinear dynamic equation, a 2nd-order Nonlinear Auto Regressive Moving Average (NARMA) task was performed, being a task that requires higher computational performance^1,25,26^; the NARMA2 task is a time series data analysis task, as shown in Eq. (8), and is a benchmark commonly used to evaluate the computational performance of RC^24–26,50–52^:

8 $$\begin{array}{c}{y}_{t}\left(k+1\right)=0.4{y}_{t}\left(k\right)+0.4{y}_{t}\left(k\right){y}_{t}\left(k-1\right)+0.6{u}^{3}\left(k\right)+0.1.\end{array}$$

The computational performance of the redox-IGR in this task was evaluated by normalized mean squared error (NMSE) as follows:

9 $$\begin{array}{c}NMSE=\frac{1}{n}\frac{\sum_{k=1}^{n}{\left[{y}_{t}\left(k\right)-y\left(k\right)\right]}^{2}}{{\sigma }^{2}\left[{y}_{t}\left(k\right)\right]}.\end{array}$$

Here, *n* is the data length, *n* = 1100 for the training phase and *n* = 150 for the test phase.

**Figure 4:**
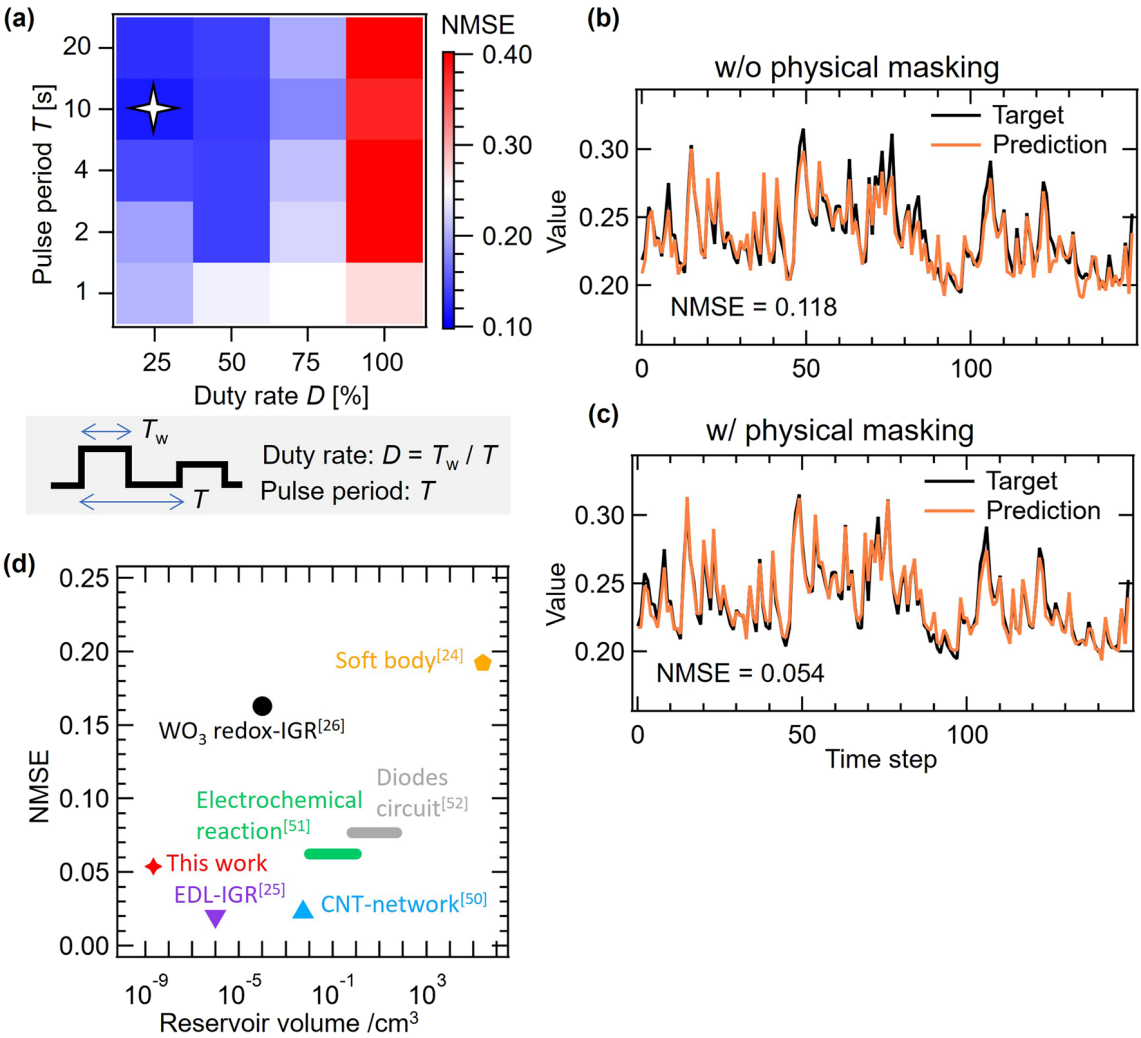
(**a**) (Upper) Relationship between NARMA2 score, pulse period T, and duty ratio *D* in the test phase. (Lower) Diagram of Pulse period and Duty rate *D*. **(b,c)** Target and prediction waveforms of the NARMA2 task w/o and w/ physical masking, respectively. **(d)** NMSEs of the NARMA2 task and reservoir volumes of various physical reservoirs. The reservoir volume of this work was calculated as the total of channel, electrolyte, and electrodes.

Figure 4a shows the relationship between the NARMA2 score, pulse period *T*, and duty rate *D*. The error decreases as *D* decreases, and is smallest at *T* = 10 s for all *D*, with the smallest error NMSE = 0.118 for the condition indicated by the star in the figure (*T* = 10 s, *D* = 25%). The target and the predicted output by IGR (test phase) under these conditions are shown in Fig. 4b. If *T* is too short, the resistance modulation of the LiCoO_2_ channel becomes small because the insertion and desertion of Li^+^ (redox) in the channel cannot follow the fast *V*_G_ pulse change. We considered that this causes the output of the device to become too similar because there is insufficient conduction modulation, which causes a decrease in computational performance. Also, if the *T* is too long, the next pulse does not input even after complete relaxation of the previous input, resulting in more similar output data, which causes the low performance observed. The neighboring virtual node diversity during the relaxation was particularly reduced. Additionally, it was observed from the color map that calculation performance improves as the duty rate decreases. In contrast, EDL-IGRs show improved calculation performance with increasing duty rate, and an IGR with a duty rate of 75% exhibited the highest performance, which led to results that were different from the previous IGR^25^. The large decrease in score (high NMSE) shown in Fig. 4a, with a duty rate of 100%, is due to the input pulse not reaching 0 V and the relaxation behavior is therefore not included in the output.

Furthermore, in order to improve computational performance within a limited number of nodes, we focused on the correlation between nodes. We reduced the calculation errors by decreasing the correlation between nodes to increase the number of nodes that are effective for computation. To reduce the correlation between the nodes, we changed the drain voltage to from a constant voltage to a triangular wave. A + 0.4 to − 0.4 V triangular wave at a pulse period 4 s was applied to drain electrode 1, which has a short channel length, and a + 0.8 to − 0.8 V triangular wave at a pulse period 10 s was applied to drain electrode 2, which has a longer channel length. As shown in Fig. 4c, the physical masking reduced the error by 72% (NMSE = 0.054) compared to when physical masking was not used. The performance of LiCoO_2_ device without physical masking is inferior to the WO_3_ device for second-order nonlinear dynamic equation task. In contrast, for NARMA2 task, which is much more difficult than the second-order nonlinear dynamic equation task in general, the performance of LiCoO_2_ is far better than the WO_3_ device regardless of with or without physical masking. Although the relatively low performance of the LiCoO_2_ device was observed for the second-order nonlinear dynamic equation task and we could not clarify the reason, we believe that the performance for the NARMA2 task is more reliable index to discuss the reservoir property. By applying physical masking, redox-IGR achieve computational performance comparable to EDL-IGR, while the physical masking does not require pre-processing to achieve significant improvements in calculation performance. This technique is a highly effective method for improving calculation performance, and can also be applied to other physical reservoirs.

Figure 4d shows the relationship between NARMA2 score and reservoir volume^24–26,50–52^. Because this device has three terminals, it is less integrated than two terminal physical reservoirs such as memristors^12,15–19,21,22,28^. However, all the devices are composed of thin films, it is relatively smaller and more integrated than most physical reservoirs reported so far, as shown in Fig. 4d.

### Relationship between the computational performance and memory capacity

We have examined the significant improvement in computational performance from the three perspectives required for reservoirs: short-term memory, nonlinearity, and high-dimensionality^1^. When performing time series data analysis tasks that are dependent on past input, it is necessary for a reservoir to have short-term memory. Short-term memory was evaluated by measuring the memory capacity (MC) of the device through a short-term memory task^1^. Said task examines how well the model can reconstruct past input data as current output. The degree of matching between the target waveform of delay length *τ* and the output waveform of the trained model can be measured by the coefficient of determination ${r}^{2}(\tau )$ shown in Eq. (10) below:

10 $$\begin{array}{c}{r}^{2}\left(\tau \right)=\frac{{\mathrm{Cov}}^{2}\left(u\left(k-\tau \right),{\widehat{y}}_{\tau }\left(k\right)\right)}{\mathrm{Var}\left(u\left(k\right)\right)\mathrm{Var}\left({\widehat{y}}_{\tau }\left(k\right)\right)}.\end{array}$$

**Figure 5:**
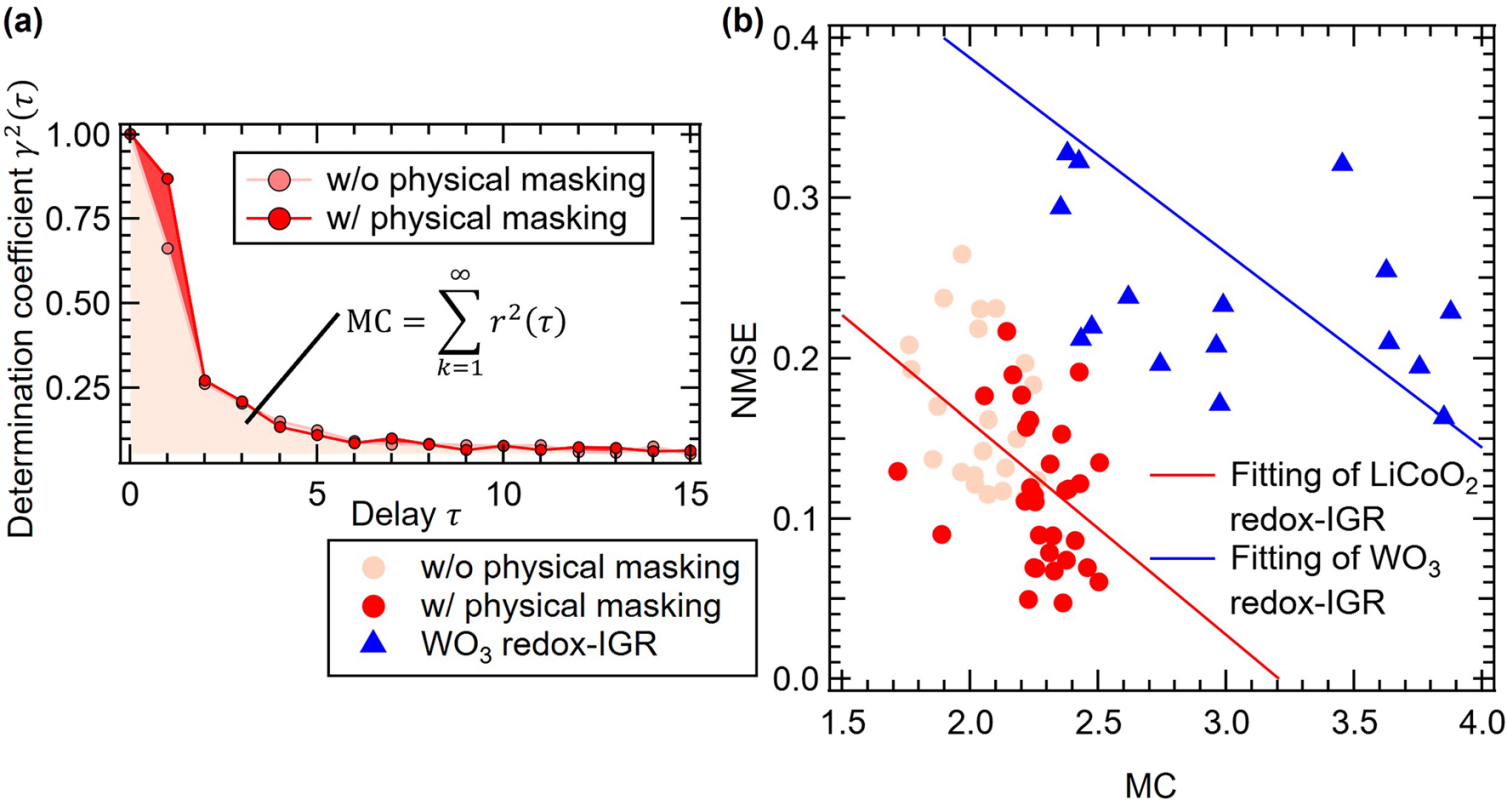
(**a**) Forgetting curves w/o and w/ physical masking. (**b**) Relationship between computational performance and MC.

Here, ${\widehat{y}}_{\tau }\left(k\right)$ represents the model output at delay length $\tau$, $\mathrm{Cov}( \cdot , \cdot )$ denotes covariance, and $\mathrm{Var}(\cdot )$ represents variance, respectively. The possible range of ${r}^{2}(\tau )$ is $0 \le {r}^{2}(\tau )\le 1$, and if the model can successfully reconstruct the delayed sequence as the model output, ${r}^{2}(\tau )$ takes a value close to 1. The variation of ${r}^{2}(\tau )$ in respect to delay length *τ* is called the forgetting curve. Forgetting curves with and without physical masking are shown in Fig. 5a. MC is defined as the area under the forgetting curve, which is described as follows:

11 $$\begin{array}{c}MC=\sum_{\tau =1}^{\infty }{r}^{2}\left(\tau \right).\end{array}$$

In regions with a long delay length $\tau$, there was almost no change in ${r}^{2}(\tau )$, but when compared to regions with a short delay length ($\tau \le 2$), ${r}^{2}(\tau )$ increased when a physical mask was applied, leading to an increase in MC. Masking is known to improve interactions between virtual nodes, and is accompanied by increasing reservoir size^49^. Because the upper limit of MC is determined by reservoir size, the physical masking in the study increases MC, and this increase in MC led to an increase in the computational performance of the device.

Figure 5b shows the relationship between the computation performance and MC. The downward-sloping relationships between NMSE and MC for both LiCoO_2_ and WO_3_ redox-IGR, approximated by the two straight lines, evidences that increased MC leads to reduced NMSE, which means improvement in the computational performance of a reservoir^26^. It is also shown that MC increases with the application of physical masking under all experimental conditions, and the NMSE of NAMRA2 is greatly reduced in LiCoO_2_ redox-IGR. Although LiCoO_2_ redox-IGRs are inferior to WO_3_ redox-IGRs in terms of MC, the NMSE is smaller. This deviation from said tendency indicates that the high computational performance achieved in this study is attributed not only to MC, but also from to other requirements (nonlinearity and/or high dimensionality)^1^.

### Relationship between computational performance and nonlinearity

**Figure 6:**
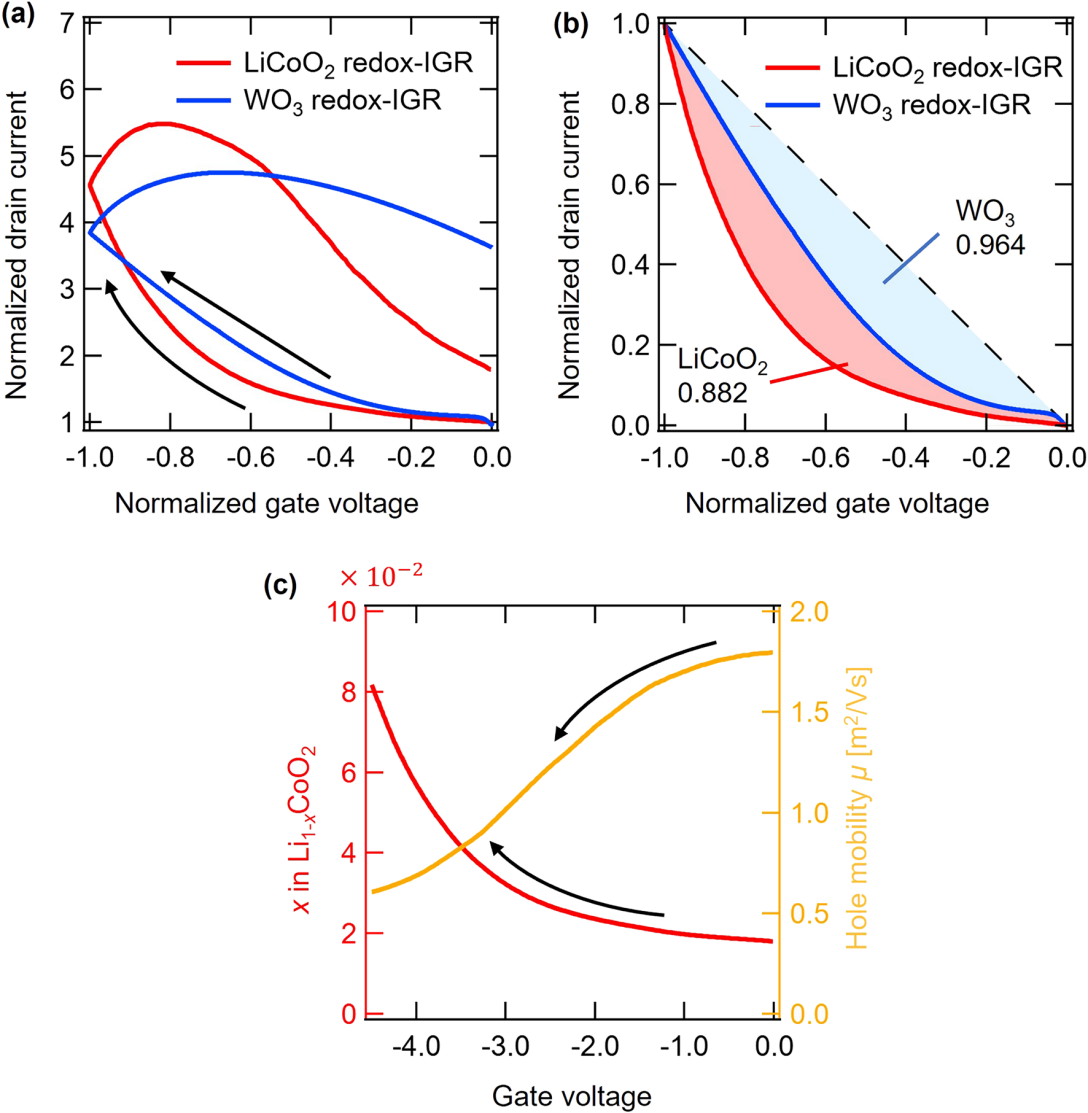
(**a**) Comparison of normalized *I*_D_ – normalized *V*_G_ curve of the LiCoO_2_ redox-IGR and WO_3_ redox-IGR. (**b**) Comparison of Nonlinearity in *I*_D_–*V*_G_ curve. (**c**) *x* in Li_1-*x*_CoO_2_ and hole mobility change of LiCoO_2_ in *V*_G_ sweep.

Nonlinearity is an element required by a reservoir to enable it to transform non-linear time-series input data, which is not linearly separable into a linearly separable state. Further, strong nonlinearity improves computational performance by diversifying the output and increasing the expressive power of the model^1^. We performed *V*_G_ sweep measurements in order to investigate the nonlinearity, on–off ratio, and reversibility. Figure 6a compares the normalized *I*_D_-normalized *V*_G_ curves of a LiCoO_2_ redox-IGR and a WO_3_ redox-IGR^26^. The on–off ratio of *I*_D_ is larger for the LiCoO_2_ redox-IGR than for the WO_3_ redox-IGR, which indicates a higher response to *V*_G_. Focusing on the normalized *I*_D_ value at the end of the hysteresis curve, LiCoO_2_ redox-IGRs are closer to the initial current value than WO_3_ redox-IGRs, which means that LiCoO_2_ has better charge–discharge reversibility than WO_3_, and the device behavior is less likely to change even if a pulse voltage is repeatedly applied during calculation. This is quite reasonable if one considers an irreversible Li^+^ trapping into a WO_3_ matrix during redox cycles, as has been reported recently^37–39^. It further means that the LiCoO_2_ redox-IGR has high reproducibility as a time-series input–output converter, and the same time-series output can be obtained for the same time-series input, without depending on random initial conditions. Such good reproducibility in the LiCoO_2_ redox-IGR leads to the achievement of the echo state property, which is an important property required for reservoirs, regardless of whether they are simulated or physical reservoirs^2^.

To evaluate nonlinearity, we compared the correlation coefficients of the *I*_D_-*V*_G_ curve and the linear line (*I*_D_ =  − *V*_G_). The correlation coefficient, calculated by Eq. (12), is a measure of how linear the *I*_D_-*V*_G_ curve is; if the linearity is high, the correlation coefficient is close to 1, and if the linearity is low, the correlation coefficient is close to 0.

12 $$\begin{array}{c}R\left({x}_{i},{y}_{i}\right)=\frac{\sum_{i=1}^{n}\left({x}_{i}-\overline{x }\right)\left({y}_{i}-\overline{y }\right)}{\sqrt{\sum_{i=1}^{n}{\left({x}_{i}-\overline{x }\right)}^{2}}\sqrt{\sum_{i=1}^{n}{\left({y}_{i}-\overline{y }\right)}^{2}}}.\end{array}$$

In Eq. (12), *n* represents the total number of data points, ${x}_{i}$ and ${y}_{i}$ are the value of normalized drain current and the value of the linear function, respectively. $\overline{x },\overline{y }$ are the average of each value. Regarding the correlation coefficient with a linear line and an *I*_D_–*V*_G_ curve, the correlation coefficients for the LiCoO_2_ redox-IGR and WO_3_ redox-IGR are 0.882 and 0.964, respectively, which shows that the LiCoO_2_ redox-IGR has a more nonlinear change than the WO_3_ redox-IGR (Fig. 6b). Nonlinearity is one of the main functions required of reservoirs in nonlinear transformations of time series input data, and it is known that the higher nonlinearity of systems can increase their computational performance^1^. We attribute the observed performance improvement to the strong nonlinearity of the resistance modulation in the LiCoO_2_ redox-IGR, evidenced by the *I*_D_–*V*_G_ curve.

In order to consider the origin of the nonlinearity, we further analyzed the electrical characteristic of the LiCoO_2_ redox-IGR. Figure 6c shows variation in *x* in Li_1−*x*_CoO_2_ and hole mobility with respect to *V*_G_, which are derived from the *I*_D_–*V*_G_ and *I*_G_–*V*_G_ curves. *x* showed nonlinear variation in the range from 0.02 to 0.08, which is relatively close to that found in stoichiometric LiCoO_2_. On the other hand, hole mobility is also nonlinearly changed, from 1.8 × 10^–3^ to 5.0 × 10^–4^. Near the stoichiometric region, the mechanism of hole transport in LiCoO_2_ was reported to be variable-range hopping, which is characteristic of Anderson type insulator–metal transition, and the mobility observed in the present study is consistent with such report^53,54^. The origin of the strong nonlinearity of the LiCoO_2_ redox-IGR is attributed to nonlinear changes of both *x* and hole mobility.

### Relationship between computational performance and high dimensionality

In addition to MC and nonlinearity, another important condition required for reservoirs is high dimensionality^1^. High dimensionality facilitates pattern recognition in the readout section by mapping time-series input data to a high-dimensional space. For physical reservoirs, it is important how the number of effective nodes is increased while maintaining low correlation among a limited number of nodes. High dimensionality can be evaluated by the number of effective nodes used in the calculation, which in this case was evaluated by the correlation coefficient $r\left({X}_{i},{X}_{j}\right)$ between each node, using the following equation,

13 $$\begin{array}{c}r\left({X}_{i},{X}_{j}\right)=\frac{{\sum }_{k=1}^{L=150}\left({X}_{i}\left(k\right)-\overline{{X }_{i}}\right)\left({X}_{j}\left(k\right)-\overline{{X }_{j}}\right)}{\sqrt{{\sum }_{k=1}^{L=150}{\left({X}_{i}\left(k\right)-\overline{{X }_{i}}\right)}^{2}}\sqrt{{\sum }_{k=1}^{L=150}{\left({X}_{j}\left(k\right)-\overline{{X }_{j}}\right)}^{2}}}.\end{array}$$

**Figure 7:**
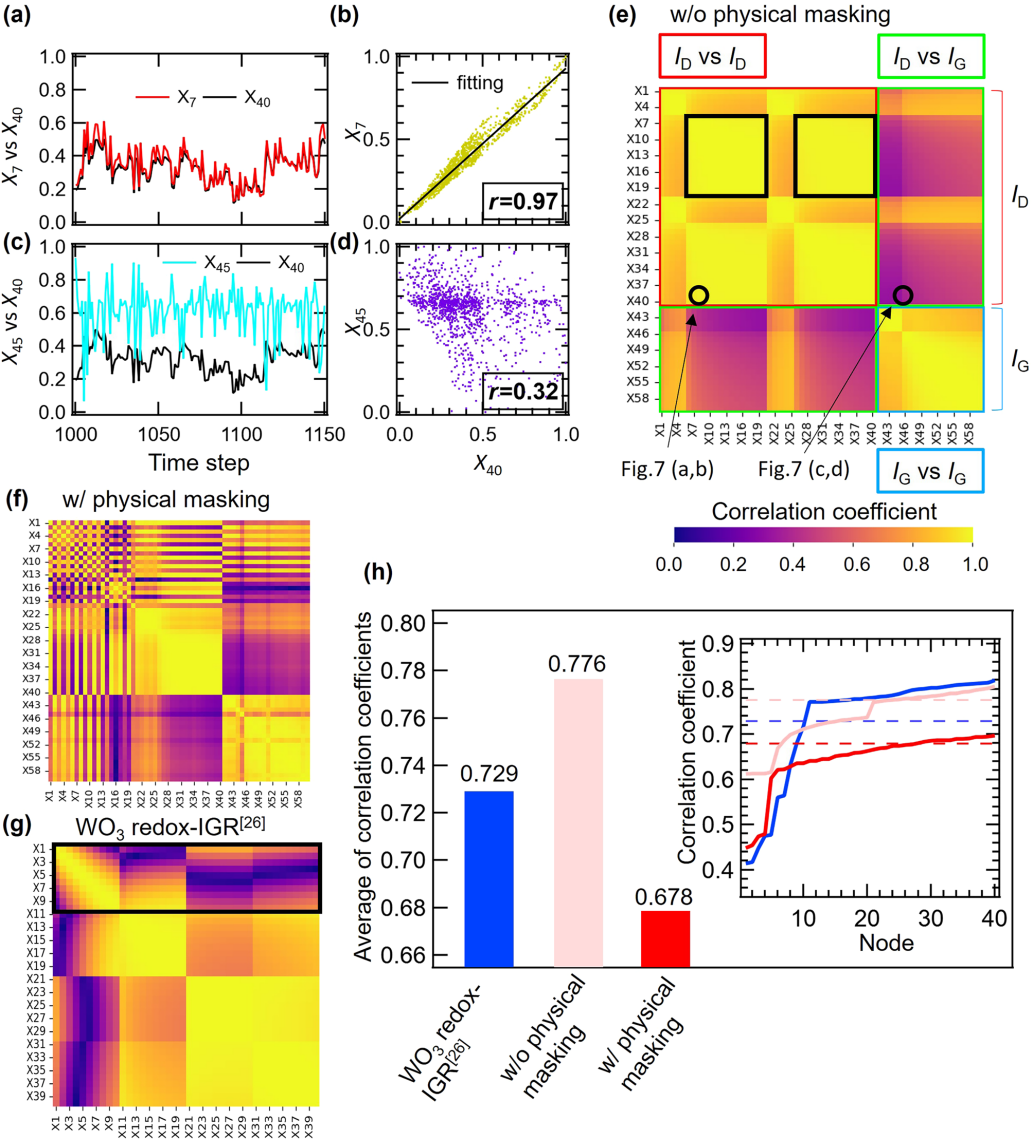
(**a**) *X*_7_ for *I*_D_ (red line) and *X*_40_ for *I*_D_ (black line) wave form. (**b**) Scatter plot between *X*_7_ and *X*_40_ with high correlation (*r* = 0.97). (**c**)* X*_45_ for *I*_D_ (blue line) and *X*_40_ for *I*_D_ (black line) wave form. **(d)** Scatter plot between *X*_45_ and *X*_40_ with low correlation (*r* = 0.32). **(e–g)** Correlation coefficient heatmap of w/o and w/ physical masking LiCoO_2_ redox-IGR and WO_3_ redox-IGR. **(h)** Distribution and average of correlation coefficients.

Here, ${X}_{i}$ and *L* are the reservoir state of node *i* and the data length, respectively. The correlation coefficient is a measure of how similar each node is; if the similarity is high, the correlation coefficient is close to 1, and if the similarity is low, the correlation coefficient is close to 0. Figure 7a shows the reservoir state wave form for node 7 (*X*_7_(red line)) and node 40 (*X*_40_(black line)), obtained from *I*_D_ response. The *X*_7_ and *X*_40_ waveform have almost the same shape, and Fig. 7b shows that they are very strongly correlated, with a correlation coefficient of 0.97. This indicates that the two node states are almost identical, and the reservoirs are not very expressive. On the other hand, the *X*_45_ and *X*_40_ waveforms shown in Fig. 7c are very different, with a low correlation coefficient of 0.32 (Fig. 7d). The large difference in the shape of the node states makes the reservoirs more expressive and reduces the error in the tasks^26^.

Figure 7e−g shows a color map of the correlation coefficients between the nodes of LiCoO_2_ redox-IGR and WO_3_ redox-IGR^26^. The results of WO_3_ redox-IGR device are shown as a reference for comparison with the performance of LiCoO_2_ redox-IGR in the study^26^. In a LiCoO_2_ redox-IGR, nodes 1 to 20 correspond to the data obtained from the *I*_D1_, nodes 21 to 40 correspond to the *I*_D2_, and nodes 41 to 60 correspond to the *I*_G_. In WO_3_ redox-IGR, nodes 1 to 20 correspond to the data obtained from the *I*_D_, and nodes 21 to 40 correspond to the *I*_G_^26^. Areas with low correlation coefficients are shown in dark colors, while areas with high correlation coefficients are shown in light colors. Figure 7e shows that the correlation coefficient is particularly low during the pulse intervals (*V*_G_ = 0 V) when the drain and gate currents are relaxing (regions *X*_7_ to *X*_20_ and *X*_27_ to *X*_40_ in Fig. 7e), which is different from Fig. 7g where the correlation coefficient is low during the application of pulse in WO_3_ redox-IGR (*X*_1_ to *X*_10_ in Fig. 7g). When comparing Fig. 7e,f, it can be seen that physical masking enhances the diversity of reservoir states and decreased the overall correlation coefficient that was suggested in Fig. 2c, mainly the correlation coefficient between nodes obtained from *I*_D1_, and that physical masking significantly decreased the NARMA2 score (from NMSE = 0.189 to NMSE = 0.054).

The average values of the correlation coefficients between nodes are listed in order of lower correlation coefficients in right-hand panel of Fig. 7h, while the dotted line is the average value of all nodes. The left-hand part of Fig. 7h shows the average of the correlation coefficients of the LiCoO_2_ redox-IGR (with and without physical masking) and the WO_3_ redox-IGR, which are 0.776, 0.678 and 0.729, respectively^26^. The distribution of correlation coefficients and the average values of correlation coefficients in Fig. 7h indicate that the higher dimensionality, achieved by decreasing the correlation between nodes through the application of physical masking, led to significant improvement in IGR performance.

## Conclusions

We developed a redox-IGR using a (104) oriented LiCoO_2_ thin film so as to overcome the low performance exhibited by previously reported redox-IGR, which is caused by irreversible Li^+^ trapping in the WO_3_ channel^26^. The subject LiCoO_2_ redox-IGR utilizes the reversible resistance change that results from the redox reaction (LiCoO_2_ ⟺ Li_1−*x*_CoO_2_ + *x*Li^+^ + *x*e^−^) accompanying the insertion and desertion of Li^+^. In addition to the use of LiCoO_2_, a physical masking technique was developed to improve performance, which it does by time-multiplexing of the input without any extra pre-processing calculation burden. The computational performance of the subject LiCoO_2_ redox-IGR was evaluated by performing a second-order nonlinear transformation task and a NARMA2 task. The prediction error was reduced by 72% and the operation speed was increased by 4 times compared to a WO_3_ redox-IGR^26^. The reason for said improvements was investigated in terms of the three aspects that are generally required for RC: short-term memory, nonlinearity, and high dimensionality. Short-term memory was evaluated by determining the MC through a short-term memory task. Although we confirmed that computational performance increased with increasing MC, the MC of the LiCoO_2_ redox-IGR was smaller than that of a WO_3_ redox-IGR, suggesting that factors other than MC had a significant impact on the high computational performance. Nonlinearity was evaluated by comparing the *V*_G_ sweep curves of redox-IGRs. From such sweep curves, we found that the LiCoO_2_ redox-IGR showed a strong nonlinear resistance change with respect to *V*_G_. High dimensionality was evaluated by determining the correlation coefficient between the nodes, and it was found that LiCoO_2_ redox-IGR had more independent nodes with low correlation coefficients, which led to high computational performance. Neuromorphic computing is of great importance in overcoming the high-power consumption of exhibited by current AI devices. In particular, physical RC has huge potential for realizing excellent compatibility with high computing performance, low power consumption, and small device volume. Our results indicate a promising way to develop high performance neuromorphic circuits consisting not only of nanoionic devices^55–67^, but also of a wide range of nanomaterials and nanoarchitectonic systems^59–73^.

## Methods

### Readout network ridge regression training

When solving time series transformation tasks such as the Second-order nonlinear dynamic equation task and NARMA2 tasks shown in Figs. 3 and 4, the readout network of the IGR is trained by ridge regression. The reservoir state vector ${\varvec{x}}\left(k\right)$ is acquired from IGR (*I*_D1_, *I*_D2_, *I*_G_), and the reservoir output $y\left(k\right)$ is the linear combination of the readout weight vector ${\varvec{w}}$ and ${\varvec{x}}\left(k\right)$**,**

14 $$\begin{array}{c}y\left(k\right)=w\cdot x\left(k\right).\end{array}$$

Here, ${\varvec{w}}=\left(b,{w}_{1},{w}_{2},\dots ,{w}_{N}\right)$**,** ${\varvec{x}}\left(k\right)={\left(1,{X}_{1}\left(k\right),{X}_{2}\left(k\right),\dots ,{X}_{N}\left(k\right)\right)}^{\mathrm{T}}$ and $N$ is a reservoir size, respectively. The cost function $J\left({\varvec{W}}\right)$ in the ridge regression is defined by Eq. (15) as:

15 $$\begin{array}{c}J\left({\varvec{W}}\right)=\frac{1}{2}\sum_{k=1}^{T}{\left[{y}_{t}\left(k\right)-y\left(k\right)\right]}^{2}+\frac{\beta }{2}\sum_{i=0}^{N}{\omega }_{i}^{2}.\end{array}$$

Here, $T,\beta$ and ${y}_{t}\left(k\right)$ are the data length in the training phase, the ridge parameter and the target output of the task generated by Eqs. (5) and (8), respectively. In this study, reservoir calculations were performed for all tasks with $T=890$ and $\beta =2\times {10}^{-3}$. The readout weight $\widehat{{\varvec{W}}}$ that minimizes the cost function $J\left({\varvec{W}}\right)$ is given by following equation:

16 $$\begin{array}{c}\widehat{{\varvec{W}}}={\varvec{Y}}{{\varvec{X}}}^{\mathrm{T}}{\left({\varvec{X}}{{\varvec{X}}}^{\mathrm{T}}+\beta {\varvec{I}}\right)}^{-1}.\end{array}$$

Here, ${\varvec{Y}}=\left[{y}_{1}\left(t\right),{y}_{2}\left(t\right),\dots ,{y}_{t}\left(T\right)\right],{\varvec{X}}\left[{\varvec{x}}\left(1\right),{\varvec{x}}\left(2\right),\dots ,{\varvec{x}}\left(T\right)\right]$, and ${\varvec{I}}\left[\subseteq {\mathbb{R}}^{(N+1)\times (N+1)}\right]$ are the target output vector, the reservoir state matrix, and the identify matrix, respectively.

### Supplementary Information

Supplementary Information.

## Data Availability

All data generated or analyzed during this study are included in this published article (and its Supplementary Information files).
