# Supplementary material for: Redox-based ion-gating reservoir consisting of (104) oriented LiCoO2 film, assisted by physical masking
Source: Sci Rep. 2023 Nov 29;13:21060. doi: 10.1038/s41598-023-48135-z (PMC10687094; doi:10.1038/s41598-023-48135-z)
Supplement: Supplementary file 1 — Supplementary Information. [file 41598_2023_48135_MOESM1_ESM.pdf]

Supplementary Information

Redox-Based Ion-Gating Reservoir Consisting of (104) Oriented $LiCoO_2$ Film, Assisted by Physical Masking

Kaoru Shibata[1,2#], Daiki Nishioka[1,2#], Wataru Namiki[1], Takashi Tsuchiya[1,2*], Tohru Higuchi[2], and Kazuya Terabe[1]

[1]Research Center for Materials Nanoarchitectonics (MANA), National Institute for Materials Science (NIMS), 1-1 Namiki, Tsukuba, Ibaraki 305-0044, Japan

[2]Department of Applied Physics, Faculty of Science, Tokyo University of Science, 6-3-1 Niijuku, Katsushika, Tokyo 125-8585, Japan

[#] Equally contributed

* Email: TSUCHIYA.Takashi@nims.go.jp

Fitting parameters

Changes in drain and gate currents when a single pulse of gate voltage is applied in Figure 1(g) was fitted with Eq. (S1). Fitting parameters are shown in Table S1. y_0, A and t_0 are constants related to the conductance, magnitude of conductance variation, and time offset, respectively.

$$f(t) = y_0 + Aexp\left(\frac{-(t-t_0)}{\tau}\right) \tag{S1}$$

Table S1. Fitting parameters

	Drain current	Gate current
y_0	$3.8743 \times 10^{-6} \pm 1.91 \times 10^{-9}$	$2.2926 \times 10^{-10} \pm 2.82 \times 10^{-11}$
A	$1.7645 \times 10^{-7} \pm 7.21 \times 10^{-9}$	$3.3297 \times 10^{-9} \pm 9.26 \times 10^{-11}$
τ	0.29349 ± 0.0381	0.6294 ± 0.0399
t_0	1283.5	1284

Comparison with V_G masking

Figure S1(a) shows a schematic diagram of the input without masking, pretreatment V_G masking, and physical masking to compare the masking applied to $LiCoO_2$ redox-IGR. To compare the effect of physical masking and V_G masking on computational performance, we solved a second-order nonlinear dynamics equation task by a redox-IGR when two applying two signal streams into one electrode, and the same single stream into the gate and drain electrodes. Applying two inputs as one input to a single

electrode is general masking which is the pretreatment of input as shown in Figure 2(a)(II), S1(a)(II). With a constant V_D of 0.4 V, the V_G pulse shown in Eq. (6) multiplied by a stepped triangular wave with a range of -1 to 1, a period of $T/4$ was input as V_G. The prediction error was 5.38×10^{-4} which was better than the case without physical masking (prediction error: 7.93×10^{-4}), but worse than the case with physical masking (prediction error: 3.19×10^{-4}), as shown in Figure S1(b). The predicted and target waveforms are shown in upper panel of Figure S1(c). Although the higher dimensionality and nonlinearity of the I_G were stronger when masking was applied to the V_G, which may be the reason for the smaller error, masking to the V_D by physical masking resulted in a smaller error and did not require preprocessing of the input, confirming the effectiveness of physical masking. When the same inputs were applied to the gate and drain electrodes, the prediction error was 1.01×10^{-3}, which was worse than the case without physical masking. The predicted and target waveforms are shown in lower panel of Figure S1(c). This is because the potential difference is smaller when the same input is used for V_G and V_D, resulting in a smaller change in Li^+ than the case of without masking.

Reversibility of $LiCoO_2$ redox-IGR

To investigate the reversibility of the device, we input a triangular wave repeatedly to the device, and the drain current responses were compared at 1, 10, 100, and 1000 cycles, as shown in Figure S1(d). There is no significant change in the drain current change width or response time after 1000 pulse inputs, although the drain current slightly shifts to larger current direction during the cycle. The result supports that the device has excellent cycle stability.

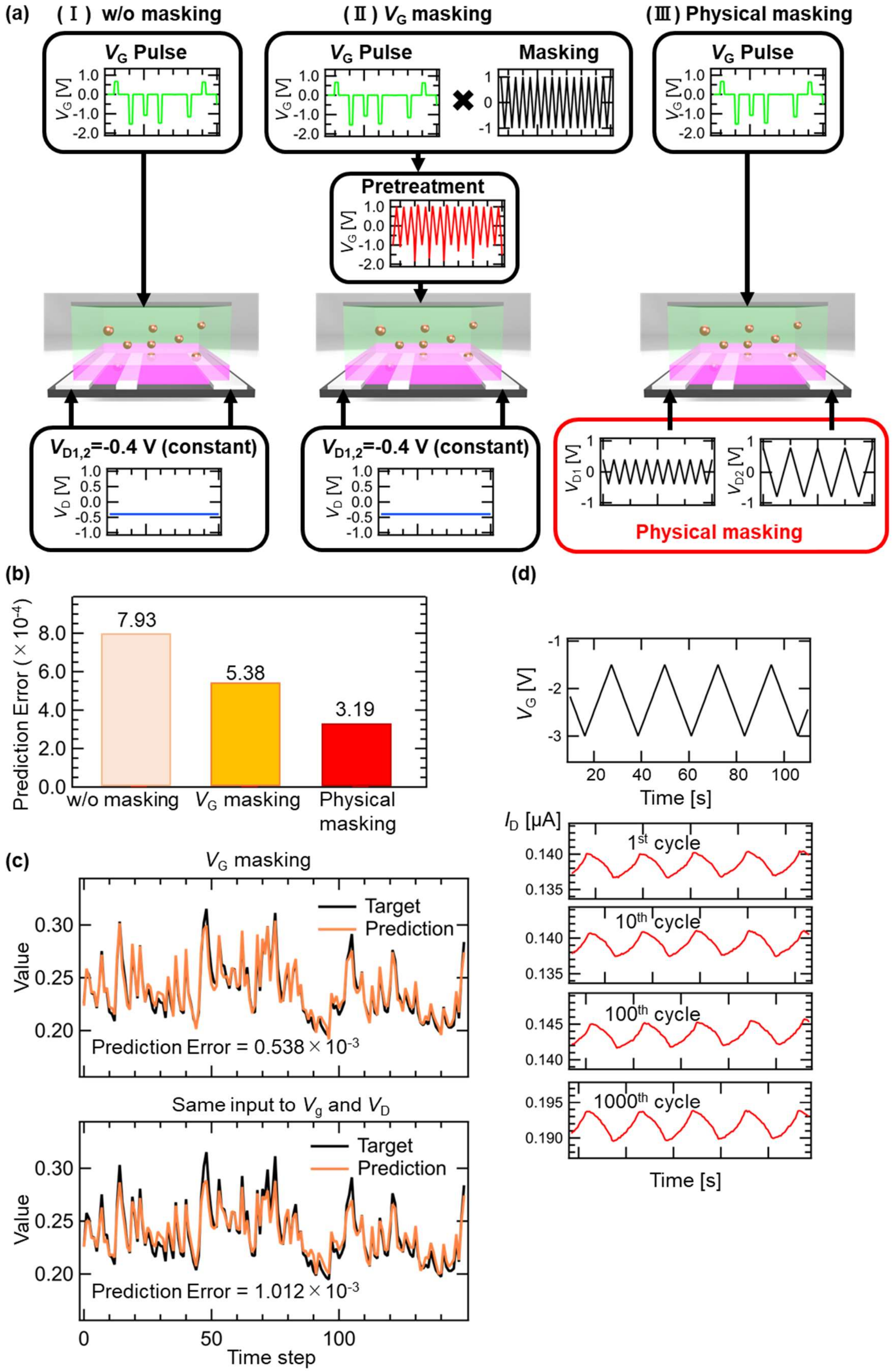

(a)
(Ⅰ) w/o masking
(Ⅱ) VG masking
(Ⅲ) Physical masking
VG Pulse
Masking
Pretreatment
VD1,2=-0.4 V (constant)
Physical masking
(b)
Prediction Error (×10-4)
7.93
5.38
3.19
w/o masking
VG masking
Physical masking
(c)
VG masking
Target
Prediction
Prediction Error = 0.538×10-3
Same input to Vg and VD
Prediction Error = 1.012×10-3
Value
Time step
(d)
VG [V]
Time [s]
ID [μA]
1st cycle
10th cycle
100th cycle
1000th cycle

Figure S1. (a) Schematic diagram of the input with (I)w/o masking, (II)pretreatment V_G masking, and (III)physical masking. **(b)** Performance comparison of w/o masking, pretreatment V_G masking, and physical masking. **(c)** Target and prediction waveform of second-order nonlinear dynamics equation task with pretreatment V_G masking and applying same input to V_G and V_D. **(d)** Input triangular wave and the drain current responses at 1, 10, 100, and 1000 cycles.
